# Aggregative Behaviour of Spiny Butterfly Rays (*Gymnura altavela,* Linnaeus, 1758) in the Shallow Coastal Zones of Gran Canaria in the Eastern Central Atlantic

**DOI:** 10.3390/ani13091455

**Published:** 2023-04-25

**Authors:** Ana Espino-Ruano, Jose J. Castro, Airam Guerra-Marrero, Lorena Couce-Montero, Eva K. M. Meyers, Angelo Santana-del-Pino, David Jimenez-Alvarado

**Affiliations:** 1 Biodiversidad y Conservación, IU-ECOAQUA, University of Las Palmas de Gran Canaria, Edf. Ciencias Básicas, Campus Universitario de Tafira, 35017 Las Palmas de Gran Canaria, Spainlorena.couce@ulpgc.es (L.C.-M.);; 2 Zoological Research Museum Alexander Koenig, 53113 Bonn, Germany; 3 Department of Mathematics, University of Las Palmas of Gran Canaria, 35018 Las Palmas, Spain

**Keywords:** *Gymnura altavela*, butterfly ray, visual census, ecology, elasmobranchs, Canary Islands

## Abstract

**Simple Summary:**

The presence of batoids in Gran Canaria, Canary Islands, Spain, in the Mid-Eastern Atlantic Ocean, is quite common depending on the species and time of year. For that reason, we examined the behaviour of spiny butterfly rays (*Gymnura altavela*) in the shallow waters of Gran Canaria, where the species’ affinity to certain beaches was analysed according to the time of year and preference for the type of ocean environment. Such knowledge is important given the lack of information available on the species that is nevertheless vital for its sufficient management and conservation.

**Abstract:**

The presence of spiny butterfly rays, *Gymnura altavela*, in waters less than 20 m deep off the Canary Islands shows marked seasonality, with relatively high abundances in the summer and autumn. Large aggregations of sometimes hundreds of individuals, primarily females, appear in specific shallow areas of the archipelago and seem to be associated with the seasonal variation in water temperature. This seasonal pattern of presence or absence in shallow areas suggests that spiny butterfly rays migrate into deeper waters or other unknown areas during the rest of the year. *G. altavela* shows sexual dimorphism; in our study, females were larger and more abundant than males, with a sex ratio of 1:18.9. The species’ estimated asymptotic length, *L*_∞_, was 183.75 cm and thus close to the common length reported for the species (200 cm). The von Bertalanffy growth constant (*k*) oscillated between 0.210 and 0.310 year^−1^, as similarly described for the species in the Western North Atlantic off the U.S. coast. From June to November, the seawater temperature oscillated between 19 and 24 °C, and massive aggregations of females occurred at 22–24 °C and in a few specific sandy beaches on the islands. Spiny butterfly rays, mostly females, show a preference for aggregating in shallow waters during summertime, probably conditionate to mating or breeding behaviour.

## 1. Introduction

Worldwide, the current situation of chondrichthyans is critical. A quarter of the species have been confirmed to be threatened, and some have lost more than 70% of their population since the 1970s [1,2]. In this context of population decline, elasmobranchs play an important role in the regulation and structuring of the marine ecosystem as key predators [3,4,5]. Some studies have suggested that the decimation of a predator population changes the relative abundance of the prey population, which consequently suggests that elasmobranchs are an important element in the marine food web, one whose reduction can initiate trophic cascades via top-down effects [3,6,7,8,9].

In general, knowledge on the biology and ecology of elasmobranchs is sorely limited, primarily because many of the species are large, migrate rapidly, range widely in depth, have low commercial value, and are challenging to keep in captivity [10]. Even within such knowledge, to date, information about elasmobranchs is highly heterogeneous [11]. It also rarely concerns deep-sea sharks and most rays and skates, which has made the status of these species’ populations rather uncertain [1,12,13,14,15].

Most such species of rays and skates inhabit coastal waters and are subject to significant anthropogenic pressure, hence their high representation among threatened species [1,16]. According to the Food and Agriculture Organization of the United Nations (2014) [17], global catches of the species decreased by nearly 20% between 2003 and 2012 [18]; however, that decrease may be far higher than indicated, for catches of the species are generally discarded, unreported, or misidentified [1]. According to Dulvy et al. (2000) [19], the decline in elasmobranchs is clearly reflected in the structure of bentho-demersal ecosystems. However, there is not enough information about the life cycles and parameters related to the distribution, longevity, reproductive periods, or age and size at maturity of many of the species to be able to realistically assess the status of their populations [20]. Regarding such data limitations and needs, the Scientific, Technical, and Economic Committee for Fisheries (2017) [3,21] has noted that the current data requirements and sampling efforts of the EU fisheries data collection programme on rays and skates cannot sufficiently provide robust estimates of various parameters (e.g., maturity, commercial catch composition, sex ratios, and indices of abundance) necessary for stock assessment and management. A case in point is the spiny butterfly ray, *Gymnura altavela* [22], a seasonally common ray in the shallow waters of the Canary Islands [23,24] that has been classified as critically endangered in the Mediterranean and Europe as well as endangered worldwide [25,26,27]. Nevertheless, the lack of data from other areas within the range of *G. atlavela* makes it difficult to define the conservation status of its population [27].

As a species of the Gymnuridae family, *G. altavela* exhibits demersal behaviour and inhabits the sandy, muddy bottom of shallow waters in tropical temperate regions [28] on both sides of the Atlantic Ocean, including in the Mediterranean and Black Seas [28,29,30,31]. The typical disc width of the largest species of the Gymnuridae family is 200 cm, and it can reach 60 kg in weight [32,33]. The coloration on its dorsal side allows the species to camouflage itself with the sandy bottom, where it spends a great part of the day resting [34].

Within the Canary Islands in the Eastern Central Atlantic, the spiny butterfly ray is as common as other benthic elasmobranchs [24,35,36] and, at the population level, seems to have a much better conservation status than in other European waters, including the Mediterranean Sea [37]. Two possible explanations for the species’ seemingly favourable conservation status in the Canary Island archipelago are that it is not targeted by local artisanal fisheries and that trawling activity has been banned in the archipelago since 1986. Although no fisheries there target any ray species, some such species are nevertheless accidentally caught in the gillnets and handlines of artisanal and recreational fisheries [38,39,40,41].

Given the limited ecological and biological information that can be obtained using fishery-related surveys, citizen science programmes afford a valuable opportunity to provide insights into the ecological distribution and population structure of critically endangered species. A good example of the importance of citizen science programmes as a source of information is the conservation programme for the angel shark, *Squatina squatina*, in the Canary Islands [35,42]. Even so, the information provided by such programmes has many limitations for highly migratory species, including the periods that they can be observed in shallow coastal waters when sighted by divers. In this case, visual underwater censuses have emerged as an effective method of generating detailed biological, ecological, and behavioural data that can be used in management and conservation programmes [43]. Among the most highlighted behavioural patterns of several ray species, seasonal massive aggregations reported in many parts of the world make them highly vulnerable to human coastal activities (e.g., tourism and pollution). The environmental and/or social reasons behind this behaviour nevertheless remain somewhat unclear [44,45].

Against that background, in our study, we aimed to generate knowledge about the seasonal presence of spiny butterfly rays in shallow coastal areas. We especially sought data for an a priori assessment of the population structure and status of the species in the Canary Islands region that could be useful to create a baseline for the development of effective conservation measures for such a highly vulnerable fish species.

## 2. Material and Methods

### 2.1. Data Collection

**Figure 1:**
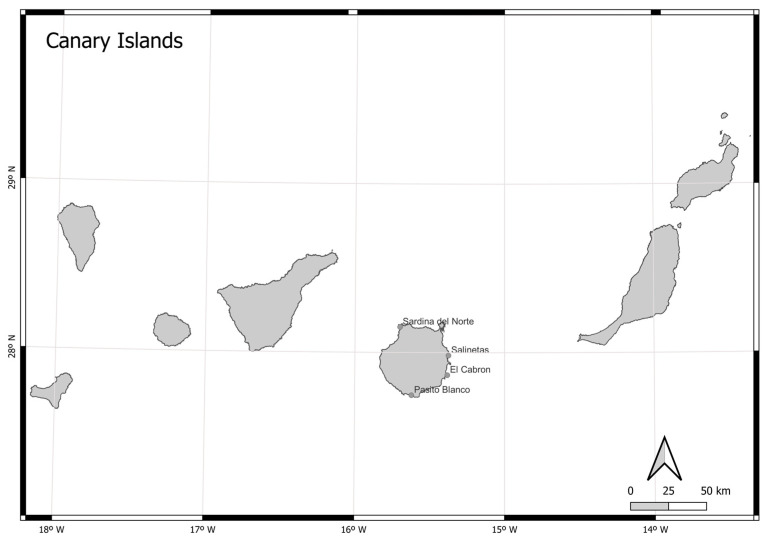
Geographical situation of the Canary Islands and the sampled areas (i.e., Sardina del Norte, Salinetas, El Cabrón, and Pasito Blanco).

Our study was conducted around the island of Gran Canaria, located in the centre of the Canary Island archipelago, Spain, in the mid-eastern Atlantic Ocean. Biological and behavioural data of spiny butterfly rays were obtained via visual census conducted from March 2018 to March 2020 near four different beaches (Sardina del Norte, Salinetas, El Cabrón, and Pasito Blanco) on the north-western, eastern, south-eastern, and southern sides of the island, respectively (Figure 1).

In total, 108 visual censuses—27 surveys at each sampling site—were conducted at the different beaches over a 2 year period. Due to differences in the topographic and bathymetric characteristics of the sampling sites, visual censuses at Salinetas and Pasito Blanco, which have sandy bottoms with a low slope and high turbidity, were conducted at 0–5 m deep, whereas visual censuses at Sardina del Norte and El Cabrón beaches were conducted at 5–20 m deep due to the high slope and sandy bottoms in deeper waters.

Visual censuses were conducted by three divers using snorkelling equipment in areas 0–5 m deep and diving equipment in deeper areas, all following the method described by Labrosse et al. (2002) [46]. The researchers lined up at a maximum distance of 5 m from each other, a distance that could vary due to good or bad visibility in the different areas. The described areas were sampled in their entirety, which allowed obtaining precise data about the number of individuals of each species present in each area. During each survey, all individual spiny butterfly rays found in the area were recorded, sexed, and measured in terms of total length (i.e., from the tip of the nose to the end of the tail) and disc width (i.e., along the maximal axis), both in cm, estimated by approaching the rays with a tape measure as they rested on the bottom. We also recorded whether individuals were alone, aggregated, resting, feeding, swimming, and/or showed escape behaviour or any other characteristics that could be useful for the study, including body marks. Different environmental data (e.g., seawater temperature, turbidity, sea conditions, and, if present, companion species) were recorded as well (Table 1).

Added to the data recorded by the divers during the visual censuses, data concerning spiny butterfly ray sightings were obtained from the databases of the citizen science programmes Poseidon, at the University of Las Palmas de Gran Canaria (www.programaposeidon.eu, accessed on 17 September 2018), and RedPROMAR, within the government of the Canary Islands (https://redpromar.org/sightings?region_id=3, accessed on 15 October 2021), which collects marine biodiversity monitoring data for the Canary Islands. Whereas RedPROMAR provides data from sightings from 2015 to the present, Poseidon provides such data representing 2014–2018. Both databases include the number of individuals observed and the position of the sighting. In sum, we obtained data from 434 recorded sightings of *G. altavela*.

### 2.2. Data Analysis

Data obtained from the visual censuses were analysed using R version 4.2.2 (R Core Team, 2022). Generalised additive models (GAMs) for count data (i.e., Poisson and negative binomial models), including zero-inflated models, were considered in modelling the number of observed individuals. The Akaike information criteria (AIC) was used to select the model that best fit the data, which was found to be the zero-inflated Poisson GAM. The model assumes that the probability that no ray individuals are observed (or arrive) at a beach is *p*, while the number of rays arriving at the beach, *b_i_*, in month *m* follows a truncated Poisson distribution of parameter *µ* (i.e., $P\left(N=k\right)=\frac{p{µ}^{k}}{\left({µ}^{k}-1\right)k!}$), a function of the given month and beach calculated as:

$$µ=µ\left(m,b_{i}\right)=exp$$

such that *I*(*b_i_*) = 1 if the data comes from beach *b_i_* and 0 otherwise.

Data obtained from both years sampled were standardised for joint analysis and compiled as the number of individuals recorded per square metre per minute of sampling. The data series presented a non-normal distribution (Kolmogorov–Smirnov test, *p* < 0.05); thus, nonparametric methods were used in data analyses. The growth parameters were also estimated using the von Bertalanffy growth function (VBGF; von Bertalanffy, 1938) in the R package TropFishR [47]. Growth parameters were analysed using different optimisation techniques as a function of length frequencies (i.e., 10 cm class interval), the data for which were collected monthly. The asymptotic length and the von Bertalanffy growth rate were calculated using the ELEFAN model with simulated annealing and a genetic algorithm, both as approaches to optimise the model. In the same way, the ELEFAN model with the best score fit (i.e., high *R*_n_) was selected. Based on the Powell–Wetherall method described by Wetherall et al. (1987) [48], the asymptotic length, *L*∞, was estimated.

## 3. Results

**Figure 2:**
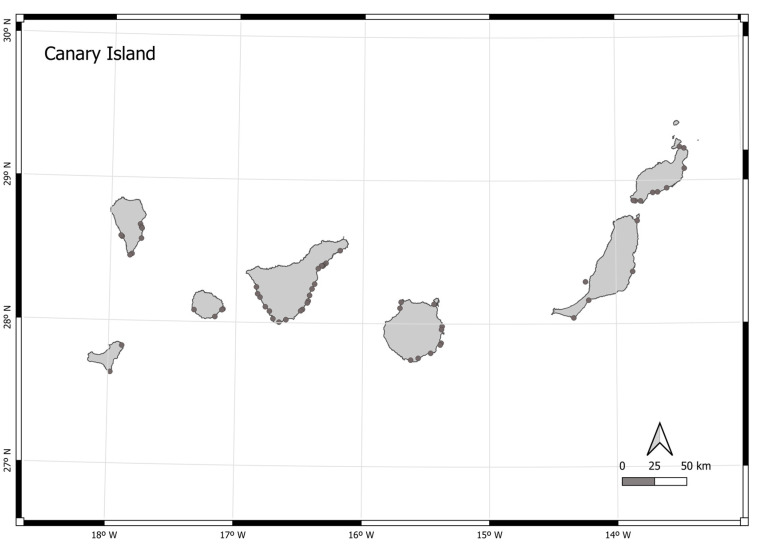
Map of the geographical situation of the Canary Islands, with areas where spiny butterfly rays were represented by grey dots.

The information available on the geographical distribution of spiny butterfly rays in the Canary Islands, according to data extracted from sighting programmes provided by citizen science databases, is highly conditioned by the assiduity of divers at certain dive sites. For that reason, the entire visual census was conducted in four areas described in citizen science data. Figure 2 shows the coastal areas of the islands where sightings are most frequent, which possibly coincide with areas of the islands where the most frequent sightings occur and/or where massive aggregations of individuals of the species occur.

**Figure 3:**
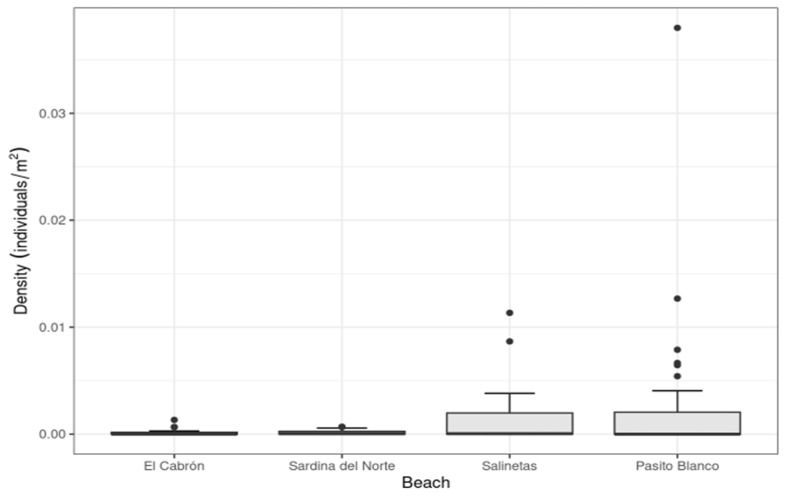
Density of spiny butterfly rays per unit of area at each beach surveyed. Black dots (out layers) represent massive aggregations out of the mean and standardized deviation.

**Figure 4:**
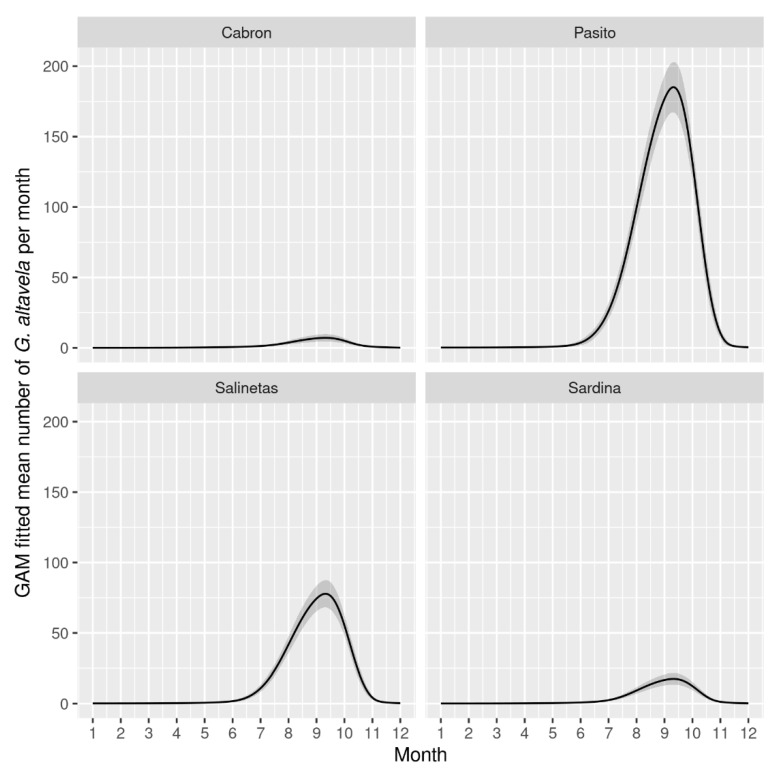
*Gymnura altavela*’s annual cycle pattern, drawn from a zero-inflated Poisson GAM (AIC = 416.9). Grey area (out layers) represent massive aggregations out of the fitted mean.

**Figure 5:**
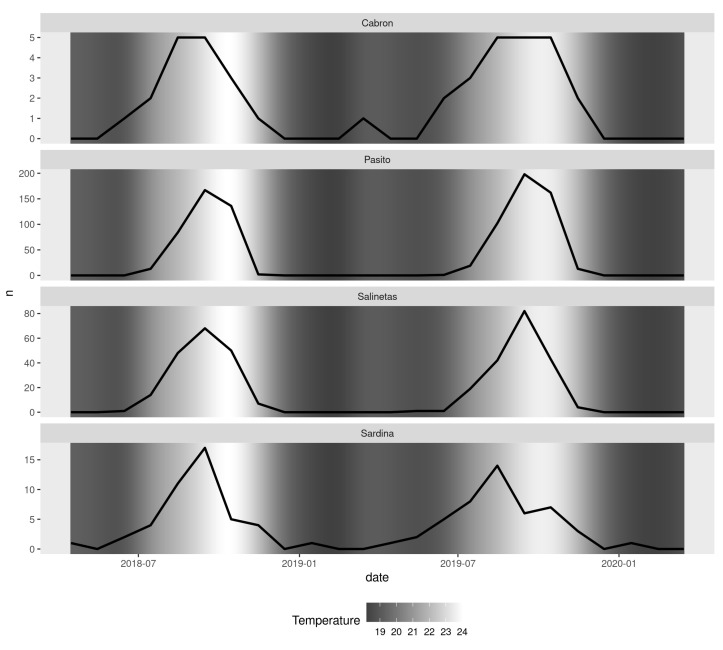
Number of spiny butterfly rays per month and year in relation to mean temperature (Spearman’s correlation = 0.81, *p* > 0.0001).

The highest number of spiny butterfly rays per square metre was recorded at Pasito Blanco beach, at the leeward side of Gran Canaria (Kruskal–Wallis ANOVA, H = 60.794, *p* = 0.047; Figure 3). Several GAMs were fitted to the number of individuals observed per beach and per month, but the zero-inflated Poisson GAM fit the data best (AIC = 416.9). This model shows an annually cycled pattern of the number of individual spiny butterfly rays, with aggregations occurring from June to November and peaking in September (Figure 4). This pattern in the seasonal presence of individual spiny butterfly rays in the extremely shallow waters of some beaches seems to be influenced by the variation in seawater temperature (Spearman’s correlation, *r* = 0.81; *p* > 0.0001, Table 2). Rays began arriving at the shallow beaches when the seawater reached 19 °C, i.e., at the beginning of summer (i.e., in June), and the number of individuals aggregating increased as the temperature reached 22–24 °C. Likewise, the aggregation decreased as the seawater decreased in temperature. Individuals began leaving the areas when the temperature fell below 22 °C in mid-autumn, i.e., at the end of October (Figure 5).

**Figure 6:**
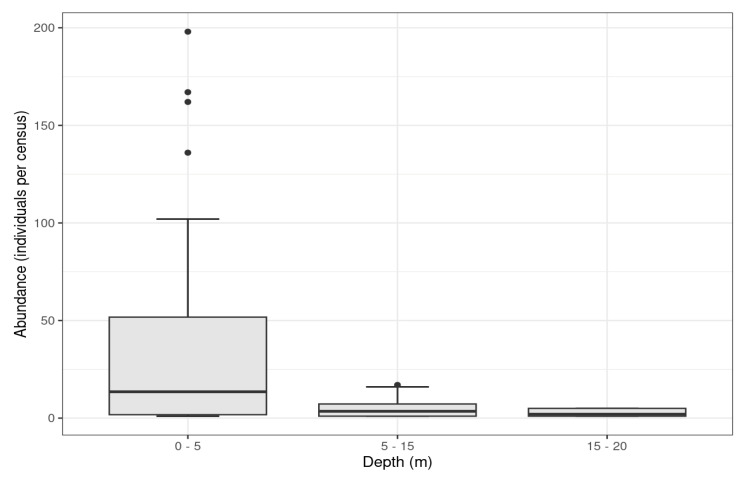
Number of individuals identified at different depths. Black dots (out layers) represent massive aggregations out of the mean and standardized deviation.

The information collected shows that during these aggregations, spiny butterfly rays are distributed in shallow waters 0–20 m deep, but the highest abundances were reported between 0 and 5 m deep, with decreases in the number of individuals as the depth increased (Kruskal–Wallis ANOVA, *H* = 12.995, *p* = 0.001; Figure 6).

Most individuals (88%) were observed while buried, resting on the sand, and very close to each other, with some partly on top of other rays and piled up in a relatively short area (e.g., approx. 10% of the total sandy area of the beaches). Few rays (12%) were observed swimming and only one individual was observed feeding.

**Figure 7:**
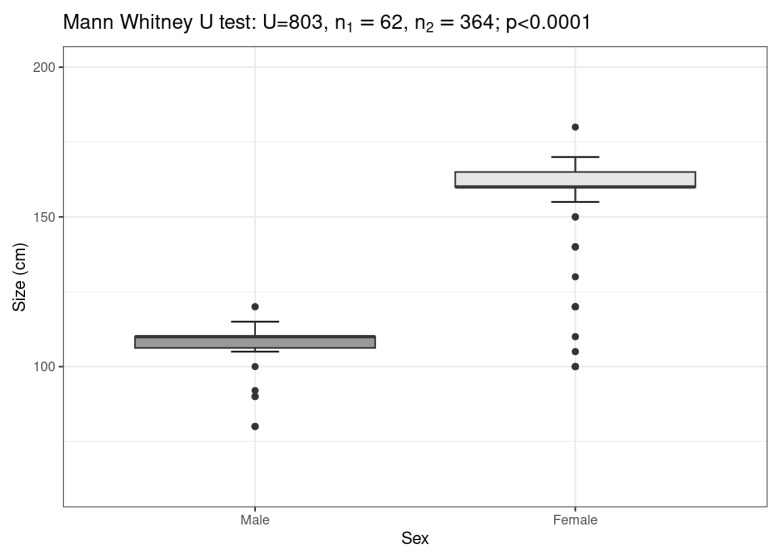
Mean total length of male and female spiny butterfly rays at Salinetas beach.

**Figure 8:**
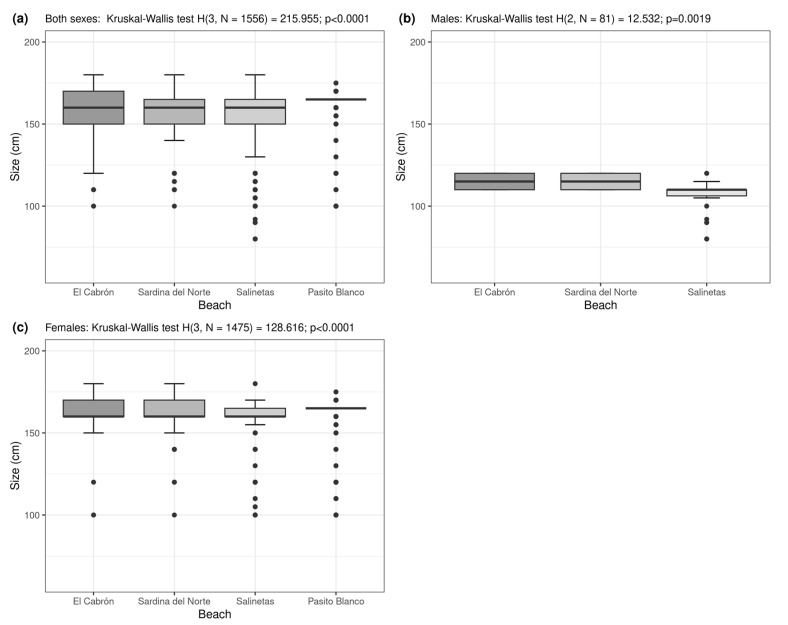
Mean total length of spiny butterfly rays in different sampled areas: (**a**) all individuals, (**b**) males, and (**c**) females.

Of the 1424 individuals counted during the sampling period, females predominated significantly, with a female-to-male ratio of 1:18.9 (χ^2^ test = 786.08, *p* < 0.0001). Males were significantly smaller than females (Mann–Whitney *U* test= 803.00, *Z* = −12.1448, *p* < 0.0001, Figure 7) and primarily observed at Salinetas to east of the island, although a few were found at Sardina del Norte. No males were reported at Pasito Blanco to the south, where the vast majority of females were observed. At the same time, significant differences in the frequency distribution of length were observed in different areas (Kruskal–Wallis ANOVA, *H* = 215.955, *p* < 0.0001, Figure 8a). The mean body size also varied between males (Kruskal–Wallis ANOVA, *H* = 12.532, *p* = 0.0019, Figure 8b) and females (Kruskal–Wallis ANOVA, *H* = 128.616, *p* < 0.0001, Figure 8c) from beach to beach, with the largest at Pasito Blanco and the smallest at Salinetas on average.

We also measured parameters of the VBGF using two ELEFAN methods: a simulated annealing algorithm and a genetic annealing algorithm. With *L∞* = 200 ± 7 cm and *k* oscillating between 0.22 and 0.23 year^−1^, the genetic algorithm showed the best goodness of fit (Table 3).

## 4. Discussion

The spiny butterfly ray, *Gymnura altavela*, seasonally aggregates in a few specific sandy beaches of the Canary Islands. The reasons why certain areas and not others are selected for these aggregations remains unknown, and beaches that seem to have similar environmental and oceanographic characteristics—beach orientation to waves or currents, sand granulometry, depth, water temperature, and food availability, among others—are not visited while others are [49]. Most likely, the presence of natural and/or artificial breakwater structures provides adequate shelter and resting areas for the rays [36]. Large aggregations of ray species have been observed at several beaches near breakwater structures on all of the islands of the archipelago. Likewise, refuging behaviour has also been observed in other rays and shark species (i.e., *Squatina squatina* or *Bathytoshia centroura*), often in physical proximity, if not contact, during regular periods of the year, not only in the Canary Islands but also in many other parts of the world [44,50,51].

In contrast, environmental factors such as seawater temperature seem to influence the presence of spiny butterfly rays in shallow waters and their aggregative behaviour near some sandy beaches. In this regard, our analysis revealed the existence of significant correlations between seawater temperature and patterns of the arrival and departure of rays from beaches. These relationships have also been considered to trigger movements and changes in the behaviour and habitat use of many elasmobranch species [35,52]. Along these lines, our GAM analyses revealed that spiny butterfly rays’ arrival at some shallow beaches is a cyclical process—they begin arriving en masse in July and disappear in mid-November—which might suggest a certain philopatry, site fidelity and/or seasonal residency. Similar tendencies have been described by Jaine et al. (2014) [53] regarding reef manta rays in a Capricorn eddy region in Australia and by Gaspar et al. (2008) and Davy et al. (2015) [54,55] regarding *Urogymnus granulatus* and *Pateobatys fai*, who exhibited seasonal residency at Orpheus Island and the Mo’orea marine reserve, respectively. However, we did not detect any relationship between the numbers of rays aggregated per beach and seawater temperature. Nevertheless, water temperature can trigger a biological process that causes the species to approach the beach at the beginning of summer [56].

The largest numbers of spiny butterfly rays, primarily females, aggregated at specific beaches in the Canary Islands during the summer and early autumn, when the seawater temperature fluctuated between 19 and 24 °C. Such aggregation could be related to reproductive events. Mull et al. (2008) [57] have proposed that water temperature (18–20 °C) plays an important role in the regulation of testosterone in round stingray (*Urobatis halleri*) males and may ultimately cue the reproduction of the species. Along those lines, Taylan et al. (2019) [22] reported a pregnant *Gymnura altavela* caught in the Aegean Sea in April 2018 that had three well-developed embryos (i.e., 28.6–30.9 cm disc width), which indicated that fecundation had probably occurred 6 months prior (i.e., around October). However, the low number of males observed at sites of aggregation in our study did not indicate a mating aggregation, at least during the day, for only one male was observed chasing a female. The absence of males could be explained by their frequency in deeper waters, offshore, or in different areas far from the sampled areas. Mating events could have also happened at night. In fact, and as observed in other species such as *Carcharhinus melanopterus* and *Tetronarce californica* [58], data suggested that, at night, the spiny butterfly rays were more active and moved further away from the shallow areas where they were found resting during the day. Observations of mating among rays and skates are rarely reported in the literature, and copulation is normally preceded by chasing, in which males bite onto the anterior portion of the females’ disc [59]. We observed some pregnant females in the areas studied, as well as the presence of newborns; thus, we cannot rule out that the aggregations of females are related with breeding seasons.

Similarly, large concentrations of individuals in very shallow waters have been described in other ray species, including *Pateobatys fai* in French Polynesia [60], *Aetobatus narinari* in the Gulf of México [61], and *Mobula alfredi* in West Papua [62]. However, significant differences arose in the abundance of spiny butterfly rays per unit area (individuals/m^2^) across the four beaches surveyed. Those differences could relate to the topography of the beach strands and the abrupt changes in the depth of the subtidal zones. Rays were more abundant in waters 0–5 m deep at beaches with less sloped bottoms and more pleasant surf zones (e.g., Pasito Blanco and Salinetas). In contrast, they were less numerous in places that gain in depth quickly and where waves break more heavily against the shore (e.g., El Cabrón and Sardina del Norte). In general, rays aggregate in deeper areas from 10 to 20 metres deep.

*G. altavela* shows sexual dimorphism, with females being larger than males, a characteristic also reported in many other skates and ray species [63,64,65,66], including *G. altavela* in the Western Atlantic [67].

Elucidating the population dynamics of *G. altavela* first requires examining the parameters of the von Bertalanffy growth equation. In that sense, the estimated *L∞* for the spiny butterfly rays in Gran Canaria waters was 200 ± 7 cm and thus similar to the common length reported for the species but far from the maximal length reported [32]. In contrast, the growth constant (*k*) that we estimated oscillated between 0.22 and 0.23 year^−1^, a growth coefficient similar to that described for other ray and skate species [68,69,70,71] and close to that reported by Parsons et al. (2018) [67] for individuals in the North Atlantic off the U.S. coast. Parsons et al. reported *k* values of 0.27 ± 0.04 for males and 0.6 ± 0.1 for females obtained from high-resolution X-ray computed tomography of the vertebral centre. These differences in the growth rhythms of spiny butterfly rays between the north-western Atlantic and the Canary Islands could be related to differences in the methodology of parameter estimation and/or with differences in environmental conditions, including the availability of food between highly productive areas and oligotrophic ones.

To be sure, extensive work is needed to obtain enough information to develop adequate strategies for conserving *G. altavela* and a management plan is required to guarantee the healthy status of its population around the Canary Islands. Implementing measures to protect shallow areas where the species seasonally aggregates to mate or breed are also crucial for their survival and future conservation.

## 5. Conclusions

Spiny butterfly rays aggregate in the shallow waters of the sandy beaches of Gran Canaria during the summer and autumn, with a peak of individuals in September. The beach of Pasito Blanco showed a significantly higher aggregation of individual rays per square metre than in the other beaches surveyed. The animals began to arrive at the beaches when the seawater temperature reached 19 °C at the beginning of summer (i.e., in June), and the aggregation of individuals increased until the temperature reached 23–24 °C and decreased as temperatures cooled, especially when the temperature reached 19 °C at the end of October. The spiny butterfly rays distributed in shallow waters 0–20 m deep but predominated at 0–5 m, thus decreasing in abundance with depth. The estimated female-to-male ratio was 1:18.9, with a significant predominance of females. Females were also significantly larger than males. Other significant differences emerged in the length of individuals between the areas observed, with rays from Pasito Blanco being the largest and those from Salinetas being the smallest on average. Among other results, the von Bertalanffy growth equation estimates were *L∞* = 183.75 cm and *k* = 0.210–0.310 year^−1^, while the total mortality rate was *z* = 0.314 year^−1^.

## Figures and Tables

**Table 1:** Data provided by observers through the website.

Information
Diving centre or person who reported the sighting
Ability to distinguish the species
Date and location of sighting
Date or season of observation
Approximate length
Sex
Presence of offspring
Number of individuals observed
Aggregated or solitary status
Behaviour (e.g., resting, hunting or mating)
Body marks or injuries (e.g., by hook or spear gun)
Ability to reidentify the reported individuals due to those body marks

**Table 2:** Correlation between the number of individual spiny butterfly rays and mean temperature.

Correlation	Var1	Var2	Corr.	*p*	Conf. Low	Conf. High
Pearson	Temperature	N	0.86	0.0000000988	0.6906081	0.9359712
Spearman	Temperature	N	0.81	0.0000017800		

**Table 3:** Growth parameters and scores obtained from ELEFANs simulated annealing (SA) and genetic annealing (GA) algorithms for *Gymnura altavela* population. The selected model was based on the highest *R*_n__max values, marked in bold.

Parameter	ELEFAN S.A.	ELEFAN G.A.
Asymptotic length (cm)	206.07	199.28
Growth coefficient *k* (yr^−1^)	0.22	0.23
Summer point oscillation (ts)	0.22	0.39
Amplitude of growth oscillation (C)	0.35	0.79
Growth performance index (Φ’)	3.96	3.95
t_anchor	0.31	0.60
Age_max	14	14
Goodness of fit (*R*_n__max) score	0.58	**0.60**

## Data Availability

The datasets generated and/or analysed during the current study are available from the corresponding author upon reasonable request.
